# Caspases and matrix metalloproteases facilitate collective behavior of non-neural ectoderm after hindbrain neuropore closure

**DOI:** 10.1186/s12861-018-0175-3

**Published:** 2018-07-31

**Authors:** Naomi Shinotsuka, Yoshifumi Yamaguchi, Kenichi Nakazato, Yudai Matsumoto, Atsushi Mochizuki, Masayuki Miura

**Affiliations:** 1 Department of Genetics, Graduate School of Pharmaceutical Sciences, The University of Tokyo, Bunkyo-ku, Tokyo, 113-0033 Japan; 2 Hibernation Metabolism, Physiology and Development Group, Institute of Low Temperature Science, Hokkaido University, Sapporo, Hokkaido 060-0819 Japan; 3 Theoretical Biology Laboratory, RIKEN, Wako, Saitama 351-0198 Japan; 4 Laboratory of Mathematical Biology, Institute for Frontier Life and Medical Sciences, Kyoto University, Sakyo-ku, Kyoto, 606-8507 Japan

**Keywords:** Apoptosis, Caspases, Matrix metalloproteases, Neural tube closure, Live-imaging

## Abstract

**Background:**

Mammalian brain is formed through neural tube closure (NTC), wherein both ridges of opposing neural folds are fused in the midline and remodeled in the roof plate of the neural tube and overlying non-neural ectodermal layer. Apoptosis is widely observed from the beginning of NTC at the neural ridges and is crucial for the proper progression of NTC, but its role after the closure remains less clear.

**Results:**

Here, we conducted live-imaging analysis of the mid-hindbrain neuropore (MHNP) closure and revealed unexpected collective behavior of cells surrounding the MHNP. The cells first gathered to the closing point and subsequently relocated as if they were released from the point. Inhibition of caspases or matrix metalloproteases with chemical inhibitors impaired the cell relocation.

**Conclusions:**

These lines of evidence suggest that apoptosis-mediated degradation of extracellular matrix might facilitate the final process of neuropore closure.

**Electronic supplementary material:**

The online version of this article (10.1186/s12861-018-0175-3) contains supplementary material, which is available to authorized users.

## Background

Neural tube closure (NTC) is a complex morphological process through which the neural plate is transformed into the closed neural tube by fusing both sides of the edge of the neural plate. NTC consists of sequential morphological events: elevation and apposition of the neural plate, contacts, fusion, and remodeling. Failure in any of these processes can lead to neural tube defects, including spina bifida and severe brain malformations like anencephaly in humans [1, 2].

NTC in mammals initiates from multiple points [1, 3]. In mice, closure 1 occurs from the cerebrospinal boundaries and proceeds bidirectionally to seal both the mid-hindbrain region and the spinal cord region. Closures 2 and 3 begin from the forebrain-midbrain boundary and the rostral end of the forebrain, respectively. The starting points and patterns of the closures vary among mouse strains, but the cranial closures are finished in most cases at the mid-hindbrain neuropore (MHNP) where closures 1 and 2 meet to form the closed brain. The tissue layer that makes initial contact differs depending on the region of closure in mice [2, 4–7]. At the mid-hindbrain regions, cells located around the boundary regions between the neural ectoderm and the non-neural ectoderm first contact the opposing edge of the neural plate in the midline. The contact of the boundary cells is mediated by zipping to close the neural tube which proceeds from a caudal to rostral direction in closure 1 and from a rostral to caudal direction in closure 2. In addition to zipping, the opposing neural plates sometimes contact at multiple points in the midbrain, leading to buttoning-up of closure [6]. Subsequent to the initial contact between the opposing neuroectoderm and the non-neural ectoderm, one continuous heterotopic (neural-non-neural) sheet generates two homophilic (neural-neural and non-neural-non-neural) tissues: the closed neural tube and the surface non-neuroectodermal sheet. Although the mechanisms of the closure have been extensively studied, little is known about the mechanisms of the remodeling after NTC.

Programmed cell death (PCD), mostly apoptosis, occurs extensively in the boundary regions and the midline where the fusion occurs, raising the possibility that cell death is crucial for the tissue remodeling in the neural tube [1]. However, the role of apoptosis in tissue fusion remains controversial. Embryos lacking caspase-3, caspase-9, or apoptotic protease activating factor-1 (Apaf-1), all of which are essential components for executing intrinsic apoptotic pathway, exhibit delayed NTC and often fail to close the MHNP or forebrain neuropore [8, 9]. Nevertheless, they can still form the closed midline in other regions of the neural tube [8, 10], suggesting that apoptosis is not essential for the zipping and buttoning-up processes in NTC. Importantly, apoptosis is frequently observed in other tissues formed by tissue fusion including the palate, heart, eyes, and body wall, and is shown to be crucial for tissue fusion partly via activating matrix metalloproteases in palate fusion [5, 11]. Thus, apoptosis and its executioner caspases may play an unidentified role in tissue remodeling during NTC. To determine whether apoptosis mediated by caspases plays any role in remodeling, more precise analyses on cellular and tissue behaviors after the completion of NTC are necessary. In this study, we found unexpected collective movement of cells located around the MHNP using live-imaging techniques, and we showed that the cell movements were affected by chemical inhibitors of caspases and matrix metalloproteases.

## Results

### Extensive cell relocation occurs after two closures meet to seal the hindbrain neuropore

**Fig. 1:**
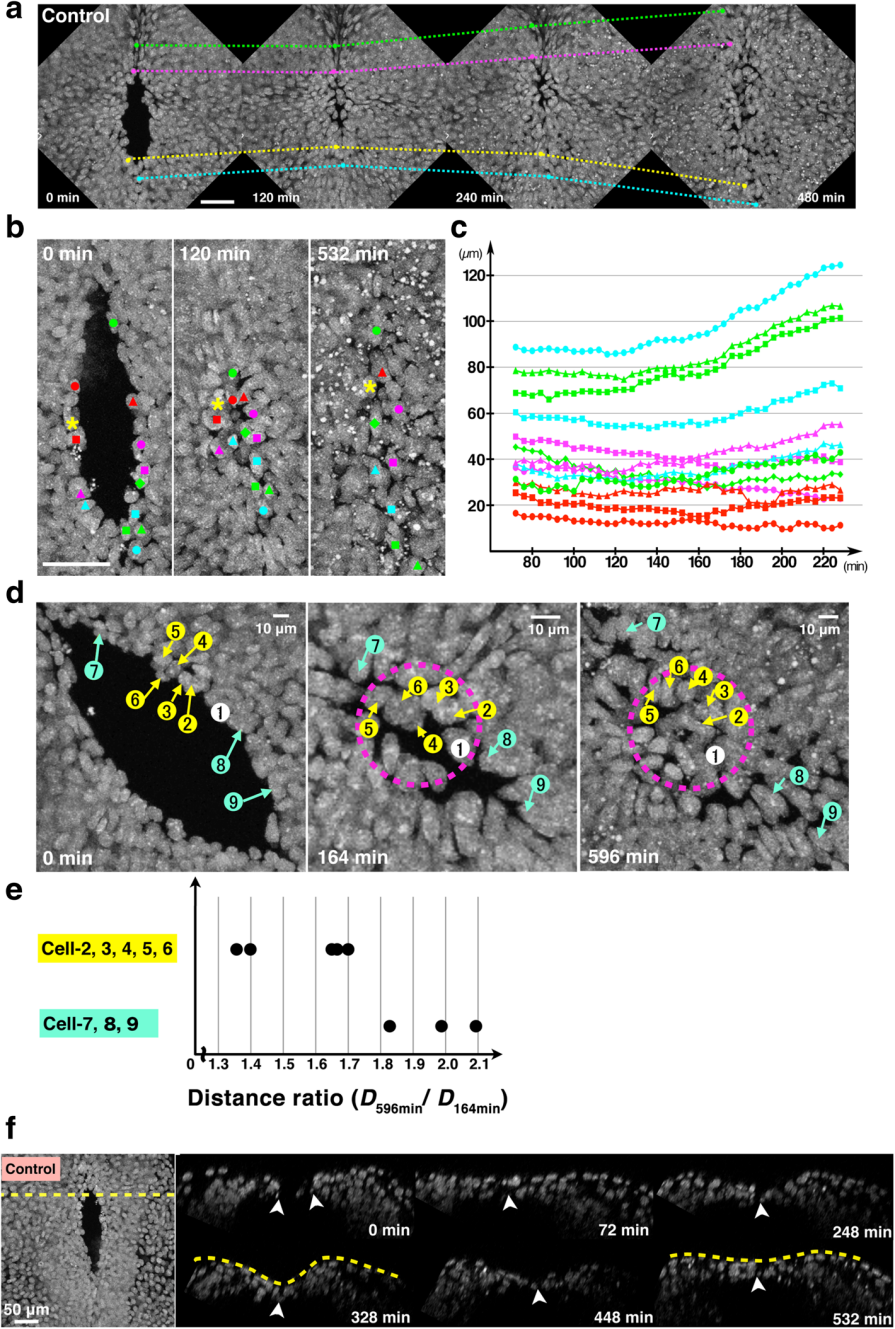
Cells on the neural ridge underwent backward movement after the completion of mid-hindbrain neuropore (MHNP) closure. **a** Time-lapse montages of the MHNP closure under live-imaging condition. Maximum z-projected images of dorsal views of the MHNP are shown. Identical cells are connected by each dotted line. **b** Magnified views of the navel in another embryo. Cells (labeled with colors) gathered near the navel and were relocated. **c** The plot of cell-cell distance between a standard cell (marked with star in (**b**)) (Y-axis) and each cell along time-points (X-axis). Each color corresponds to those of labeled cells in (**b**). **d** Representative examples of cell relocation around the MHNP in the embryo shown in (**a**). Cells on the neural ridge are labeled by numbers, and changes in their position are shown during the MHNP closure. **e** Distance ratio (D_598 min_/D_164 min_) relative to cell 1, which was selected as a standard cell within the navel. Cells located apart from the navel showed a larger distance ratio (D_598 min_/D_164 min_), indicating that those cells rapidly leave the navel regions. **f** Time-lapse montages of transverse (xz) sections reconstituted from 4D (x, y, z, t) dataset during the MHNP closure in (B-C). A dotted line in left panel (xy images) indicates the position for making xz sections shown in right panels. Scale bars: (**a**, **b**, **f**) 50 μm (**d**) 10 μm


We previously demonstrated that deficiency of apoptosis delayed the progression of MHNP closure [8]. In addition, we used live-imaging analysis with a fast scanning confocal microscope of *ex utero* culture from transgenic mice expressing a cytosolic sensor for caspase-3 activation based on FRET (SCAT3) [8, 12, 13], and noticed that the behavior of cells around MHNP after closure was different between apoptosis-deficient conditions and controls (Additional file 1: Video S1). In the control embryos, cells around the MHNP exhibited bipolar shape before the completion of MHNP closure. After closure, some cells moved away from the navel and others remained, no longer looking bipolar in shape. Apoptosis, characterized by cell fragmentation and activation of caspase-3, was observed before and after closure. When caspase activation was inhibited by a pan-caspase inhibitor Z-VAD-FMK, many cells around the MHNP were stacked in bipolar shape after completion of closure, suggesting that caspases play important roles in cell movement and changes in cell shape after closure. To understand cell movements during MHNP closure, we also performed live-imaging analysis of H2B-EGFP transgenic embryos at high resolution [14], which marked nucleus and allowed us to detect a single cell movement. To observe the final processes of MHNP closure by live-imaging analysis, we focused on embryos that already formed the MHNP but did not finish the closure in littermates. Under a live-imaging condition, the rostral and caudal closure met at the boundary of rhombomere 2 and 3 to close the neuropore (Fig. 1a, Additional file 2: Video S2). After the completion of the closure, cells at the midline were relocated as if they were released from the point where the neuropore was finally closed (Fig. 1a, each dotted line). We designated this movement as backward movement and the point where the neuropore was resolved as a navel.

Additional file 1:**Video S1.** Live-imaging of MHNP closure with or without the pan-caspase inhibitor z-VAD-FMK in SCAT3 transgenic mice. (MP4 4884 kb)



Additional file 2:**Video S2.** Live-imaging of MHNP closure in H2B-EGFP transgenic mice. (MP4 2385 kb)


To quantitatively examine the backward movement, we analyzed the movement of cells residing on the neural ridge, a circumference of the MHNP. Using 4D (x, y, z, t) dataset of live-imaging to avoid the artifact of z-projection, each cell on the neural ridge before and after the completion of NTC was tracked by the center of the nucleus labeled with H2B-EGFP at each time point. We determined the relative position of each cell by randomly defining a standard cell in a navel (labeled by a star in Fig. 1b and as 1 in Fig. 1d) and measured the distance (Dn) between the standard cell and other cells at each time point (Fig. 1c). This analysis revealed that cells moved differently depending on the original position of cells. Cells positioned near the navel (cells labeled with 2–6 in Fig. 1d) were preferentially kept at their position during imaging (Fig. 1b, c). In contrast, cells that were originally located far from the navel (cells labeled with 7–9 in Fig. 1d) showed extensive backward movement after the completion of NTC (Fig. 1b, d, e). Thus, the backward movement after the completion of NTC seemed to be correlated with the original position of cells before the completion of NTC.

### Backward movement is followed by morphological changes of the roof plate

We examined dynamics of neuropore closure using the transverse (xz) sections of the hindbrain neuropore that were reconstituted from images acquired as z-stacks of horizontal (xy) section (Fig. 1f). At the regions rostrally or caudally adjacent to the neuropore, the opposed edges of the neural folds came into contact and formed a continuous epithelial sheet. Under our imaging condition, the contact was mediated by zipping of the boundary cells located between neuroectoderm and non-neural ectoderm to form the midline (Fig. 1f, arrowheads). During this process, the edges were convoluted before the contact and zipped to form the midline groove in the newly-formed epithelial sheet. The midline groove became deeper but, subsequently, begun to resolve during the hindbrain neuropore closure in the rostral or caudal regions to the presumptive navel. As the closures proceeded toward the center of the presumptive navel, the midline groove became less evident and finally disappeared (Fig. 1f, 448 min and 532 min).

### Inhibition of caspases prevents the backward movement

**Fig. 2:**
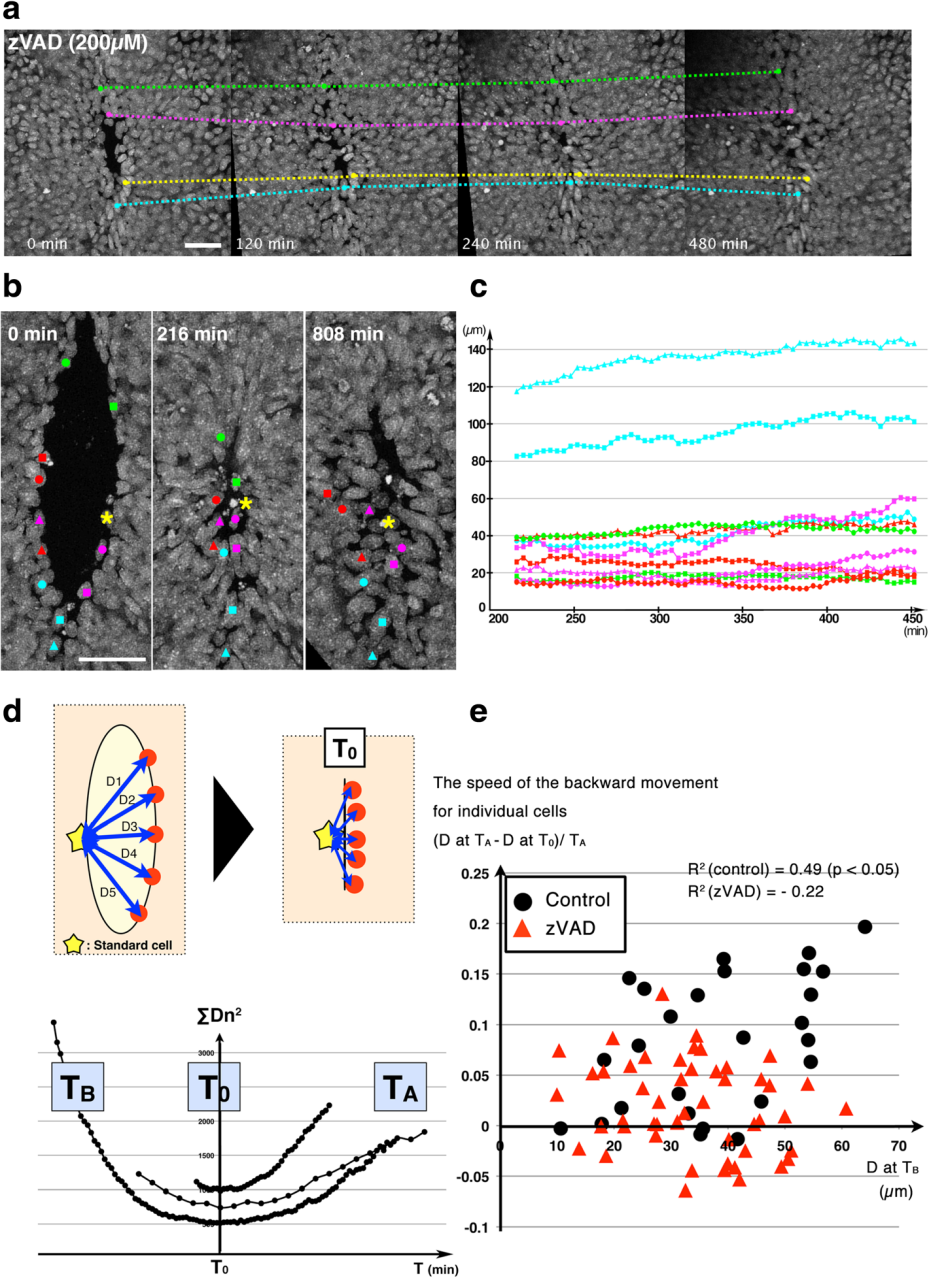
A pan-caspase inhibitor, Z-VAD-FMK, prevents cell relocation of non-neural ectodermal cells. **a** Time-lapse montages of the mid-hindbrain neuropore (MHNP) closure in the presence of a pan-caspase inhibitor, Z-VAD-FMK. **b** Magnified views of the navel in another embryo. Cells (labeled with colors) gathered near the navel failed to be released. **c** The plot of cell-cell distance between a standard cell (marked with star in (**b**)) (Y-axis) and each cell along time-points (X-axis). Each color corresponds to those of labeled cells in (**b**). **d** Schematic drawing of criteria to define T and coefficient by plotting the cell distances along times. For detail, see Results and Methods sections. **e** The speed of the backward movement was plotted against moving distance (**d**) for each cell (*n* = 27 cells from 2 control embryos, and *n* = 49 cells from 3 zVAD-treated embryos). Z-VAD-FMK treatment abolished the positive correlation between moving distances and backward movements, suggesting that caspase activation was required for the backward movement. Scale bars: 50 μm


To examine whether inhibition of apoptosis affected cell behaviors after the completion of neuropore closure, we observed embryos cultured with the pan-caspase inhibitor Z-VAD-FMK at a concentration of 200 μM which was shown to effectively inhibit apoptosis at this stage [8, 10]. This treatment inhibited the backward movement; cells located far from the navel were kept at the site when imaging started as well as cells around the navel (Fig. 2a–c, Additional file 3: Video S3). To quantify the relationship between the speed of the backward movement and the origin of the cells, we calculated the coefficient of correlation between them (Fig. 2d, e). For this analysis, time point T_0_ which indicates the timing when neuropore closure is finished, was defined as the timing when cells around the neuropore gathered most closely (Fig. 2d). Original cell positions were defined as the relative cell positions at 100 min before T_0_ (T_B_), and backward movement speed was defined as the average speed between T_0_ and at more than 200 min after T_0_ (T_A_). This analysis revealed mild positive correlation between backward speed and original cell position in normal culture condition (coefficient of correlation: 0.49 (*p* < 0.05)) (Fig. 2e, filled black circles). However, such correlation was diminished in caspase-inhibited condition (coefficient of correlation: −0.21 (*p* < 0.5)) (Fig. 2e, filled red triangles). These results suggested that cells located distant from the navel exhibited extensive backward movement in a caspase activity-dependent manner.

Additional file 3:**Video S3.** Live-imaging of MHNP closure in the presence of the pan-caspase inhibitor z-VAD-FMK in H2B-EGFP transgenic mice. (MP4 4208 kb)


### Cell shape of non-neural ectoderm changes from bipolar to polygonal after the completion of NTC

**Fig. 3:**
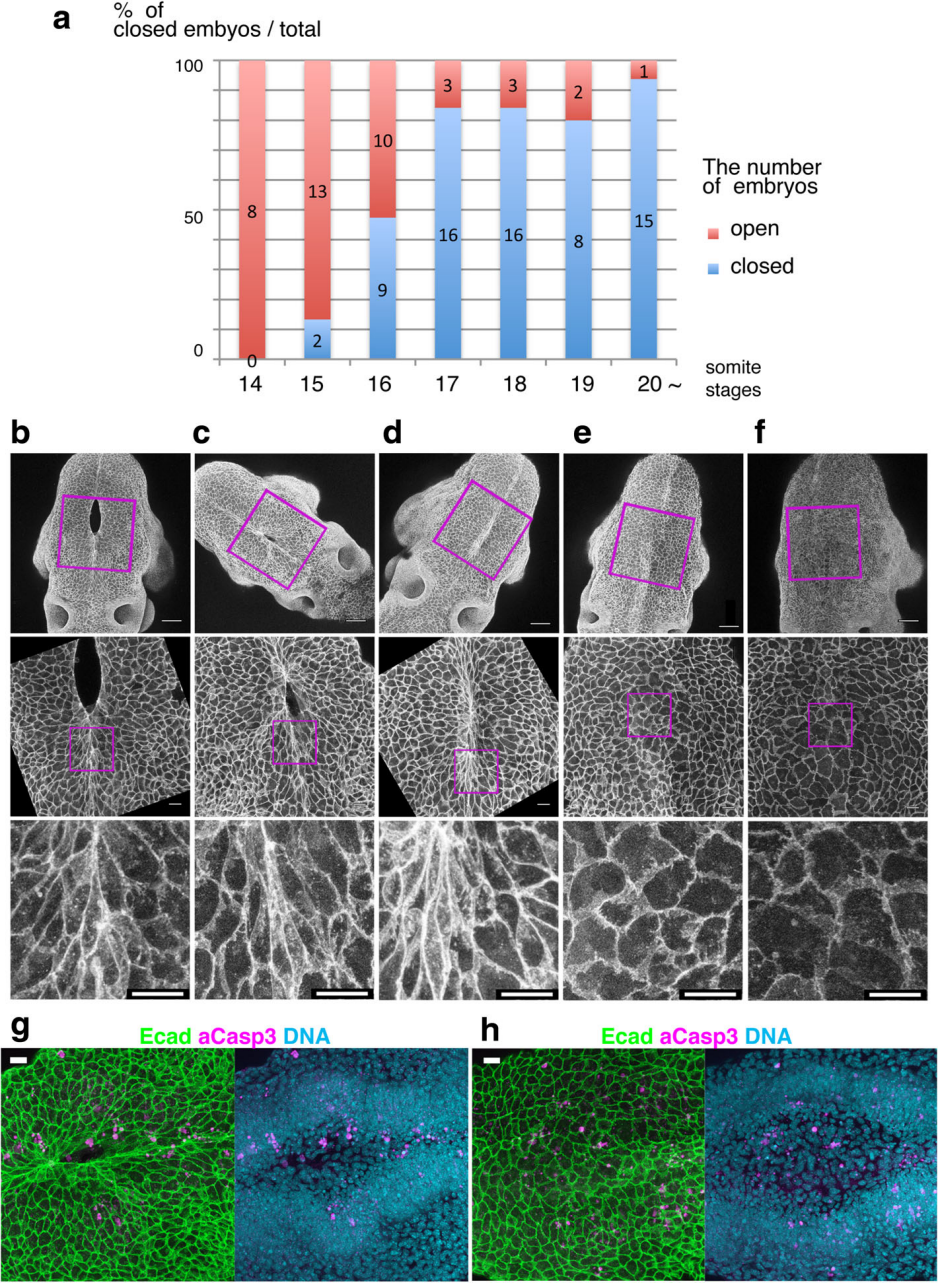
Cell shape changes in the midline after mid-hindbrain neuropore (MHNP) closure in vivo. **a** The relationship between the closing of MHNP and the somite stage in ICR strain mouse. **b**-**f** Dorsal views of embryos stained with E-cadherin (Ecad). Non-neural ectodermal cells change their shapes dramatically from bipolar to polygonal after the MHNP closure. **g**, **h** Higher manigification views of embryos (**c**, **e**) that were co-stained with Ecad, apoptotic marker active-Caspase 3 (aCasp 3), and nuclear staining (DNA). Dorsal views, left is rostral side. Scale bars: (**b**-**f**) 100 μm in upper panels, and 25 μm in (**g**, **h**) middle and (**b**-**f**) lower panels

We examined whether the backward movement occurred in utero as well as ex vitro culture. For this purpose, we traced developmental shape changes of non-neural ectodermal cells during the completion of neuropore closure. Embryos that were undergoing or just finished the hindbrain neuropore closure [somite numbers (ss)15–18] were immediately fixed after dissection and stained with E-cadherin to mark the morphology of the non-neural ectodermal cells. The hindbrain neuropore disappeared in approximately half of ss16 embryos [56% (*n* = 9/19)] (Fig. 3a). Embryos (ss15) with small neuropore exhibited the bipolar cell shapes at the midline along the rostral-caudal axis (Fig. 3b). The bipolar cell shapes at the midline were also observed in embryos (ss16) with a small or no neuropore (Fig. 3c, d), but they were not observed in embryos at later stages with no neuropore (ss17–20) (Fig. 3e, f). In those embryos with over ss18, most of the cells in the closed MHNP showed polygonal shapes (Fig. 3e, f). Thus, epithelial cells at the midline changed their shape from bipolar to polygonal after the completion of NTC in utero, which is consistent with live-image observations using a cytosolic fluorescent reporter (Additional file 1: Video S1).

### Backward movement at the midline of hindbrain is suppressed under matrix metalloprotease (MMP)-inhibited condition

**Fig. 4:**
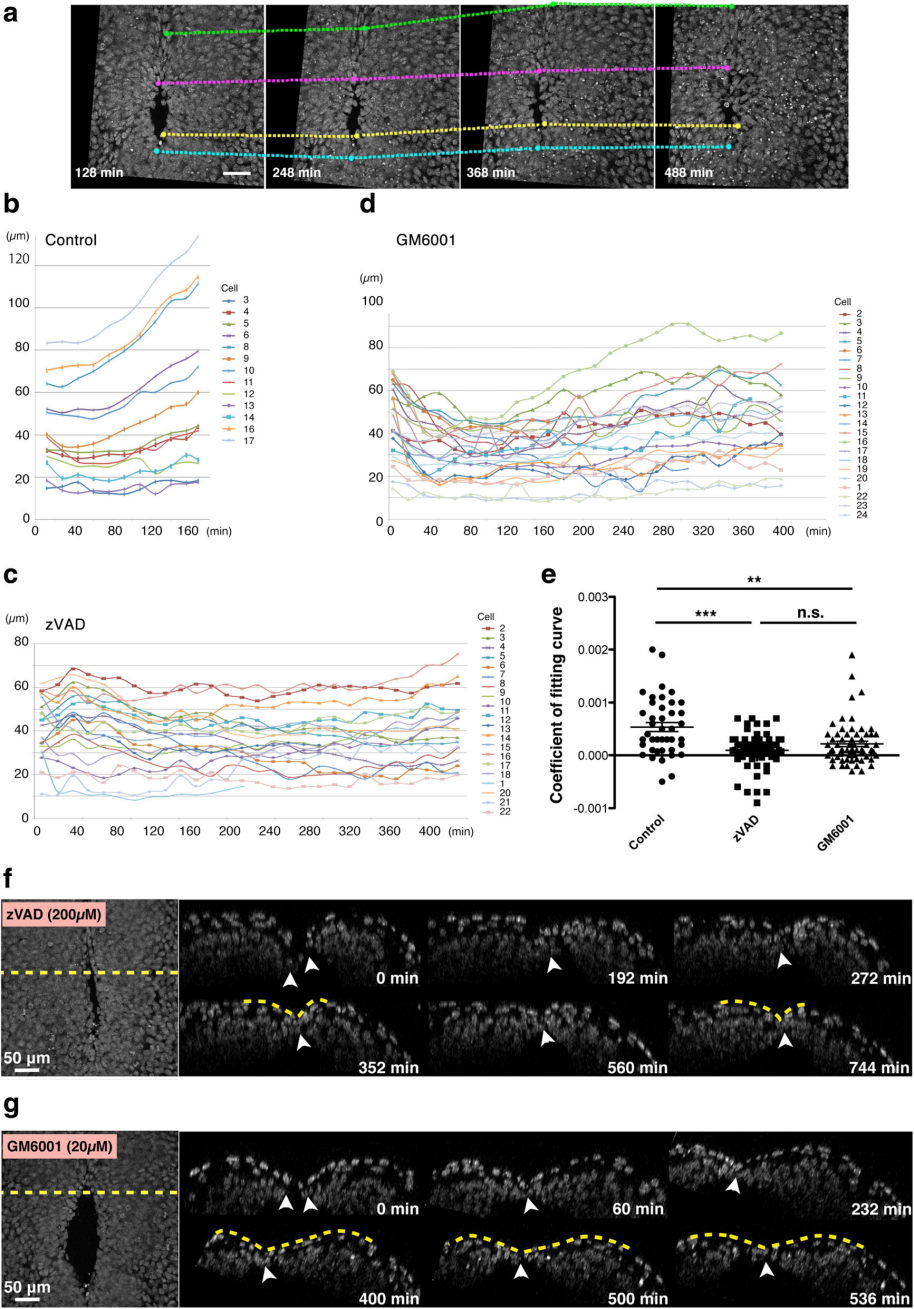
Inhibition of matrix metalloproteases (MMPs) prevents backward movement. **a** Time-lapse images of the mid-hindbrain neuropore (MHNP) closure in the presence of an inhibitor of MMPs, GM6001. **b**, **c**, **d** Examples of tracking of cells on the neural ridge during the neuropore closure of control (**b**), Z-VAD-FMK-treated (**c**), and GM6001-treated (**d**) embryos. Cells on the neural ridge were chosen evenly along the circumference of the neuropores, and their distance to a randomly-selected standard cell (Y-axis) was measured and plotted at each time-points (X-axis). Plots were created from every 16-min time-point, although images used for the tracking analysis were acquired every 4 min. Three embryos per each condition were processed for this analysis, and representative ones were presented. **e** Comparison of the speed of backward movements of cells on the neural ridge by squared term coefficients of fitting curves. Treatments with Z-VAD-FMK and GM6001 significantly reduced the speed of the backward movement (**p < 0.01, ***p < 0.001, one-way ANOVA). Each dot indicates one cell, and 41, 64, and 66 cells from three embryos per conditions were analyzed for control, Z-VAD-FMK, and GM6001, respectively. **f**, **g** Time-lapse montages of transverse (xz) sections reconstituted form 4D (x, y, z, t) dataset during the MHNP closure of Z-VAD-FMK-treated (**f**) and GM6001-treated (**g**) embryos. A dotted line in left panel (xy images) indicates the position for making xz sections shown in right panels. Scale bars: 50 μm


We addressed how apoptosis facilitated the backward movement. Part of apoptosis is observed during tissue fusion process and contributes to the smooth progression of tissue remodeling [5]. MMPs that degrade extracellular matrix act downstream of apoptosis to remodel epithelial tissues in palate fusion, one of the well-examined tissue fusion processes in mammals [11]. In fact, many apoptotic cells and fragmented apoptotic bodies were observed around the MHNP before and after closure (Fig. 3g, h). To examine if MMPs contributed to the backward movement, we treated embryos with a pan-MMP inhibitor, GM6001 [15]. We found that GM6001 also suppressed the backward movement under our live-imaging condition (Fig. 4a, Additional file 4: Video S4). To compare the effect of GM6001 and Z-VAD-FMK quantitatively, we evaluated each cell movement by creating the approximate curve on the plot obtained by measuring cell-cell length between standard cell and each cell on the neural ridge at each time point in control, Z-VAD-FMK, and GM6001 conditions (Fig. 4b, c, d). Large x squared term of approximate curve indicates that cells on the neural ridge move drastically. This analysis demonstrated that GM6001, although not so effectively as Z-VAD-FMK, suppressed the backward movement when compared to control condition (Fig. 4e). Consistent with this, the recovery of the midline groove after the MHNP closure was suppressed by Z-VAD-FMK or GM6001 treatment (Fig. 4f, g). These results suggested that MMPs as well as caspase activation were necessary for proper collective cell behavior during MHNP closure.

Additional file 4:**Video S4.** Live-imaging of MHNP closure in the presence of the pan-matrix metalloprotease inhibitor GM6001 in H2B-EGFP transgenic mice. (MP4 2570 kb)


## Discussion

Our study revealed a unique collective cell behavior, namely, the backward movement of the non-neural ectoderms covering the MHNP, using live-imaging analysis. The process consists of sequential events as follows: the chain-like bipolar boundary cells along the neural ridges gather and tackle to the navel where the neuropore is finally closed. The cells stay there for several hours and spread out as if they are released from the navel. This backward movement occurs concomitantly with shape changes of the hindbrain roof plate from concave to flat. Our findings showing that inhibition of caspases prevents the backward movement suggest its involvement in the process. It remains to be elucidated how caspases trigger the backward movement. During the period of our imaging, cell division around the neuropore were observed but not likely affected by Z-VAD-FMK or GM6001, suggesting little or minor contribution of cell division and cell crowding to the backward movement [16]. We observed many apoptotic bodies along the midline epithelial fusion site (Fig. 3g, h and Additional file 1: Video S1). This may suggest apoptosis along near the midline generates forces (apoptotic force, reviewed by [17]) for backward movement or cell shape changes for non-neural ectoderm. Backward movement seems to be coupled with bipolar to polygonal cell shape changes of non-neural ectoderm, implying that the collective effects of such cell shape changes underlie the backward movement of cells located around the midline. This could be the process releasing from high tension status of midlines to establish the stable epithelial sheet during the epithelial fusion. Although we succeeded in monitoring surface cell movement of the final process of the neuropore closure, more exhaustive analysis of cellular behaviors with deeper layers including neuroetctodermal and head mesenchymal cells will be required for fully understanding driving forces of the backward movement in future.

Inhibition of MMPs by GM6001 prevents the backward movement. This is similar to previously reported phenomenon that apoptotic cells facilitate collective cellular movements via degradation of extracellular matrix by releasing MMPs in neural crest cell (NCC) migration or a palate fusion [11, 18]; During palate fusion, opposed epithelial palate shelves are fused in the midline. First contact occurs by opposed external surface medial edge epithelium (MEE) of the palate shelves, resulting in the formation of a common medial epithelial seam (MES). MES is a transient structure, which is removed by apoptosis and extrusion of cells driven by convergence of MEE [19]. Interestingly, a pan-MMP inhibitor is effective to block the removal of MES as well as a caspase inhibitor [11], suggesting that mechanisms similar to palate fusion may be involved in the final process of NTC.

Apoptosis-deficient embryos in 129 strain-dominant backgrounds often exhibit closure defects in hindbrain, resulting in compressed neuroepithelium and insufficient brain ventricle expansion due to the leakage of cerebrospinal fluid (CSF) [9]. Thus, defective backward movement of non-neural ectoderm in the presence of caspase inhibitors might reflect insufficient brain ventricle expansion, although we were not able to assess the size of brain ventricle quantitatively, because the used imaging system did not enable us to capture whole brain regions. Driving forces of brain ventricle expansion are proposed as follows: CSF accumulation by transient occlusion of the neural tube in chicken embryos [20], cell proliferation and ion transport via neuroepithelium in zebrafish embryos [21], and epithelial relaxation mediated by myosin phosphatase regulator MYPT1 [22]. Caspase activation may regulate those processes in an apoptotic or a non-apoptotic manner, as shown by the regulation of collective cell movements in genitalia rotation or in border cell migration, respectively [23, 24]. Possible site of actions of apoptosis on the backward movement may be either the epithelial sheet including non-neural ectoderm and neuroectoderm or head mesenchyme. Head mesenchyme is crucial for the cranial NTC from the analysis of several mutant mice exhibiting defective development as well as the cranial neural tube defects (NTDs) [1]. Massive apoptotic cells in the neural ridge and around the midline may affect the behaviors of head mesenchyme.

Cells surrounding the neuropore have a bipolar shape and are aligned along the rostral-caudal axis like a chain, which differ from the underlying neuroectoderm and the adjacent non-neural surface ectoderm. These cells are located at the neural ridge, the boundary between non-neural ectoderm and neuroectoderm, termed boundary cells. The boundary cells are brought into proximity and into contact by zipping at the rhombencephalon (rostral closure 1 and caudal closure 2). In this study, we termed the final sites where the neuropore was closed as the navel and found that the navel persisted by inhibition of caspases or MMPs. We did not find abnormal structures corresponding to the persisting navel in the hindbrain of apoptosis-deficient embryos at E9.0–9.5 in vivo after the completion of NTC (data now shown). However, at later stages, we observed that ectopic cellular aggregations existed in the midbrain of apoptosis-deficient embryos around the midline, probably remnants of defective morphogenesis caused by inhibition of apoptosis (unpublished observation that will be reported elsewhere).

## Conclusions

We identified a caspase-dependent unexpected collective movement of non-neural ectodermal cells in the final process of NTC. Sealing the surface of the neural tube and the non-neural ectoderm properly would be crucial for establishing integrity of body to resist inner pressure from the rapidly growing brain ventricles. Its perturbation can lead to re-opening of the neural tube and NTDs, including excencephaly and spina bifida. Studying the final process of NTC is necessary for understanding the etiology and prevention of NTDs.

## Methods

### Mouse

H2B-EGFP transgenic mice were provided by Dr. Fujimori (NIBB, Japan) and maintained in a 129S1-C57BL6 mixed background. SCAT3 transgenic mice were generated in our laboratory [8]. ICR mice were purchased from CLEA Japan, Inc. (Tokyo, Japan). All animal work has been conducted according to the ethics guidelines of the University of Tokyo and was approved by the Ethics Committee of the University of Tokyo (Ethical Approval no. P28–11).

### Live imaging of NTC

Live-imaging of the MHNP closure using SCAT3 transgenic mice was performed as described previously [8]. To capture images of H2B-EGFP embryos, H2B-EGFP heterozygous female mice crossed with the heterozygous or homozygous male mice were killed at 8.5 days post-coitum by cervical dislocation, and the uterus was removed and placed in Opti-Minimal Essential Medium (MEM) (Invitrogen). Embryos in their yolk sac were removed from the uterus and transferred into Opti-MEM containing 10% fetal bovine serum (FBS). The yolk sac and amnion were carefully removed, while the embryo was kept on a heater, and the embryos were placed in no. 0 glass-bottom dishes (thickness of 100 μm; Matsunami Glass Ind., Ltd.) filled with Opti-MEM containing 50% of immediately centrifuged rat serum (Charles River) and 0.1% penicillin and streptomycin (Pn/St). Before the embryos were dissected, all media and dishes were pre-warmed to 37 °C. Approximately 300 μl of 2% low-melting agarose was poured into a glass-bottom dish (thickness of 100 μm; Matsunami Glass Ind., Ltd.), and a hole for sinking embryos was created at least 30 min before filling the dish with medium. Dissected embryos were sunk into the agarose holes and observed using an inverted confocal microscope (TCS SP5; Leica) equipped with a galvo stage using a high magnification, long focal length lens, 25×/ 0.98, HCx/PL FLUOTAR (Leica). During imaging, the dishes were kept in a humidified cell culture incubator with a continuous supply of 5% CO_2_/air at 37 °C (Tokai Hit Company). Enhanced green fluorescent protein (EGFP) was excited by a 488-nm argon laser using a resonant scanner (8000 Hz; Leica) and detected. The image (512 × 512 pixels) acquisition interval was 4 min, and the thickness of the z slices was 4 μm (total of 50–100 slices/each time point), depending on the experiment. For the pharmaceutical inhibition experiments, Z-VAD-FMK (200 μM final concentration; 1/1000× dilution of 200 mM stock in dimethyl sulfoxide (DMSO); Merck) and GM6001 (20 μM final concentration, 1/1000× dilution of 20 mM stock in DMSO; Merck) were used.

### Image processing

Image processing was performed using ImageJ (National Institutes of Health) as previously reported [8]. XZ transverse images were processed from raw 4D datasets with re-slice function of ImageJ. The video editing and linear adjustment of intensity for visualization were performed with ImageJ. To eliminate the XY drift in time-lapse series, the StackReg plug-in was used [25].

### Tracking and quantification of cell movements

Tracking of surface cells was performed in maximum intensity projection xy images along the z axis, and z position was automatically derived from original 3D (x, y, z) data using a program for identifying z position giving the maximum intensity at the selected xy point. We constructed a simple program to determine depth, z, from the position, (x,y), on MIP image.

Firstly we have an arrayed image stack, X[i,j,k], which means the brightness X of the (i,j) pixel on the k-th image.

MIP image can be expressed as Y[i,j] = max_k(X[i,j,k]), that is the max brightness along the 3rd axis, the depth.

In principle, we can get the depth, z, easily using this relation: Z[i,j] = argmax_k(X[i,j,k])

Thus, here is our python program, given our image files are named as ‘imagexxx.tif’.

---- python code ----.

- #import cv2
- #import numpy as np
- #import glob
- #itertools

- # load images and get an 3d arrayed data stack.
- stack = np.array([cv2.imread(fname) for fname in sorted(glob.glob(image*.tif))])

- # get the size of images
- xmx,ymx = stack.shape[0],stack.shape [1]

- # this is the array, where the solved z is placed in.
- Z = np.zeros((xmx,ymx))
- for i,j in itertools.products(range(xmx),range(ymx)): Z[i,j] = np.argmax(stack[i,j,:])

--------.

However, this Z can include some fake values, because of the noise in the original image set. Since such an fake value can be characterized as an outlier around the place of (i,j), we can detect that like following:

---- python code ----.

- # making a blurred image of Z, using the kernel with the size (s,s). Here we used s = 5.
- s = 5
- kernel = np.ones((s,s))
- blurredZ = cv2.blur(Z,kernel).astype(np.int)

- # we can detect outliers with the threshold value, th, like th = 10.
- th = 10
- outs = [i,j for i,j in zip(*np.where(np.abs(Z.astype(np.int)-blurredZ) > th))]

- # we can replace such outliers with local averaged values.
- for i,j in outs: Z[i,j] = blurredZ[i,j]

Time point when neuropore closure was finished (T_0_) was defined as the timing when cells around the neuropore gathered most closely, which was calculated by plotting total sum of squared distances among each cell (Dn) along time (∑Dn^2^) (Fig. 2d).

For determining the moving distances of cells around the neuropore, standard cells locating in the middle of the neuropore were randomly chosen at 100 min before T_0_ (T_B_), and the distance (Dn) between the standard cell and each cell around the neuropore was calculated using 3D (x, y, z) data. The speed of the backward movement was calculated as the average speed between T_0_ and T_A_ (more than 200 min after T_0_). Plots of distances between the standard cells and each cell along time were used for creating approximate curves (Fig. 2b-d), and squared term coefficients were compared in control (*n* = 41 cells from 3 embryos), Z-VAD-FMK-treated (*n* = 47 cells from 4 embryos), and GM6001-treated embryos (*n* = 39 cells from 3 embryos) (Fig. 4e). Statistical analysis was performed using ANOVA in Prism (GraphPad).

### Whole-mount immunohistochemistry

Whole-mount staining of embryos was performed as previously described [9]. E9.0–9.5 embryos collected from the uterus in cold phosphate-buffered saline (PBS) were immediately fixed with 4% paraformaldehyde (PFA) for 3 h, washed three times, and blocked with a blocking buffer (20% immuno-block/10% donkey serum/TBST (Tris-buffered saline (TBS) with 0.1% Triton X-100) for 1 h. Embryos were incubated with anti-E-cadherin (1/500× dilution; Takara) and anti-active Caspase-3 (1/500 × dilution; CST) overnight at 4 °C with gentle shaking. After eight washes in TBST, embryos were incubated with 2 μM Hochest 33,452, anti-rat IgG antibody conjugated with Cy5, and anti-rabbit IgG antibody conjugated with Cy3 (Jackson ImmunoResearch Inc.) overnight at 4 °C, washed again, and observed with a confocal microscope (TCS SP5) using HCX PL FLUOTAR 10× 0.3 NA and HCX APO FLUOTAR 10× 0.4 NA dry objectives (Leica).

## Abbreviations

NTC: Neural tube closure
NTD: Neural tube defect
PCD: Programmed cell death
MHNP: Midbrain-hindbrain neuropore
MMPs: Matrix metalloproteases
